# Changes and tracking of bone mineral density in late adolescence: the Tromsø Study, Fit Futures

**DOI:** 10.1007/s11657-017-0328-1

**Published:** 2017-04-08

**Authors:** Ole Andreas Nilsen, Luai Awad Ahmed, Anne Winther, Tore Christoffersen, Anne-Sofie Furberg, Guri Grimnes, Elaine Dennison, Nina Emaus

**Affiliations:** 1 Department of Health and Care Sciences, UiT The Arctic University of Norway, 9019 Tromsø, Norway; 2 Division of Neurosciences, Orthopedics and Rehabilitation Services, University Hospital of North Norway, Tromsø, Norway; 3 Department of Community Medicine, UiT The Arctic University of Norway, 9019 Tromsø, Norway; 4 Division of Internal Medicine, University Hospital of North Norway, 9019 Tromsø, Norway; 5 Endocrine Research Group, Department of Clinical Medicine, The Arctic University of Norway, 9019 Tromsø, Norway; 6 MRC Lifecourse Epidemiology Unit, Southampton, UK; 7 Victoria University, Wellington, New Zealand

**Keywords:** Bone mass, Bone development, Tracking, Adolescence, Areal bone mineral density, DXA

## Abstract

***Summary*:**

Areal bone mineral density (aBMD) predicts future fracture risk. This study explores the development of aBMD and associated factors in Norwegian adolescents. Our results indicate a high degree of tracking of aBMD levels in adolescence. Anthropometric measures and lifestyle factors were associated with deviation from tracking.

**Purpose:**

Norway has one of the highest reported incidences of hip fractures. Maximization of peak bone mass may reduce future fracture risk. The main aims of this study were to describe changes in bone mineral levels over 2 years in Norwegian adolescents aged 15–17 years at baseline, to examine the degree of tracking of aBMD during this period, and to identify baseline predictors associated with positive deviation from tracking.

**Methods:**

In 2010–2011, all first year upper secondary school students in Tromsø were invited to the Fit Futures study and 1038 adolescents (93%) attended. We measured femoral neck (FN), total hip (TH), and total body (TB) aBMD as g/cm^2^ by DXA. Two years later, in 2012–2013, we invited all participants to a follow-up survey, providing 688 repeated measures of aBMD.

**Results:**

aBMD increased significantly (*p* < 0.05) at all skeletal sites in both sexes. Mean annual percentage increase for FN, TH, and TB was 0.3, 0.5, and 0.8 in girls and 1.5, 1.0, and 2.0 in boys, respectively (*p* < 0.05). There was a high degree of tracking of aBMD levels over 2 years. In girls, several lifestyle factors predicted a positive deviation from tracking, whereas anthropometric measures appeared influential in boys. Baseline z-score was associated with lower odds of upwards drift in both sexes.

**Conclusions:**

Our results support previous findings on aBMD development in adolescence and indicate strong tracking over 2 years of follow-up. Baseline anthropometry and lifestyle factors appeared to alter tracking, but not consistently across sex and skeletal sites.

## Introduction

Norway has one of the highest reported incidences of hip fractures [1]. Areal bone mineral density (aBMD) is strongly associated with fracture risk. aBMD levels in the elderly are a result of peak bone mass (PBM) achieved during growth and subsequent bone loss [2]. Adolescence is characterized by massive skeletal changes due to rapid modeling and remodeling [3]. About 40% of bone mass are accumulated around the 4 years of peak height velocity (PHV) during puberty and about 90% by the age of 18 [4, 5]. These rapid changes generate both opportunities and vulnerabilities related to future bone health. Previous studies indicate that one standard deviation increase in bone mass at the end of skeletal maturation decrease future fracture risk by as much as 50% [4]. This makes maximization of the genetic potential for bone mass acquisition a strategy for prevention of osteoporosis and fragility fractures later in life. The clinical importance of this concept depends on the degree of tracking or stability of bone mineral status from younger years into adulthood [6]. Early preventive measures can be employed if there is a high correlation between bone mass levels in the younger years and later in life. Studies report that high aBMD in athletes or low aBMD due to deficits may persist into adulthood [7, 8]. Previous population-based longitudinal studies demonstrate strong tracking of aBMD from childhood to skeletal maturity [9–13]. The degree of tracking from adolescence into adulthood is, however, unclear [14–16]. Potential variation in tracking into adulthood and inconsistent evidence [10–12] calls for attention to predictors of deviation from tracking in late adolescence. The objectives of this population based longitudinal study were (1) to describe the changes in bone traits over 2 years in Norwegian adolescents aged 15–19 years, (2) to explore tracking of aBMD status over 2 years, and (3) to identify baseline anthropometric measures and lifestyle factors associated with deviation from tracking. It is our hypothesis that participants mainly remain in their original aBMD quartile between the ages of 15 and 19 years of age and that baseline predictors of positive deviation from tracking can be detected.

## Methods

### Subjects

**Fig. 1:**
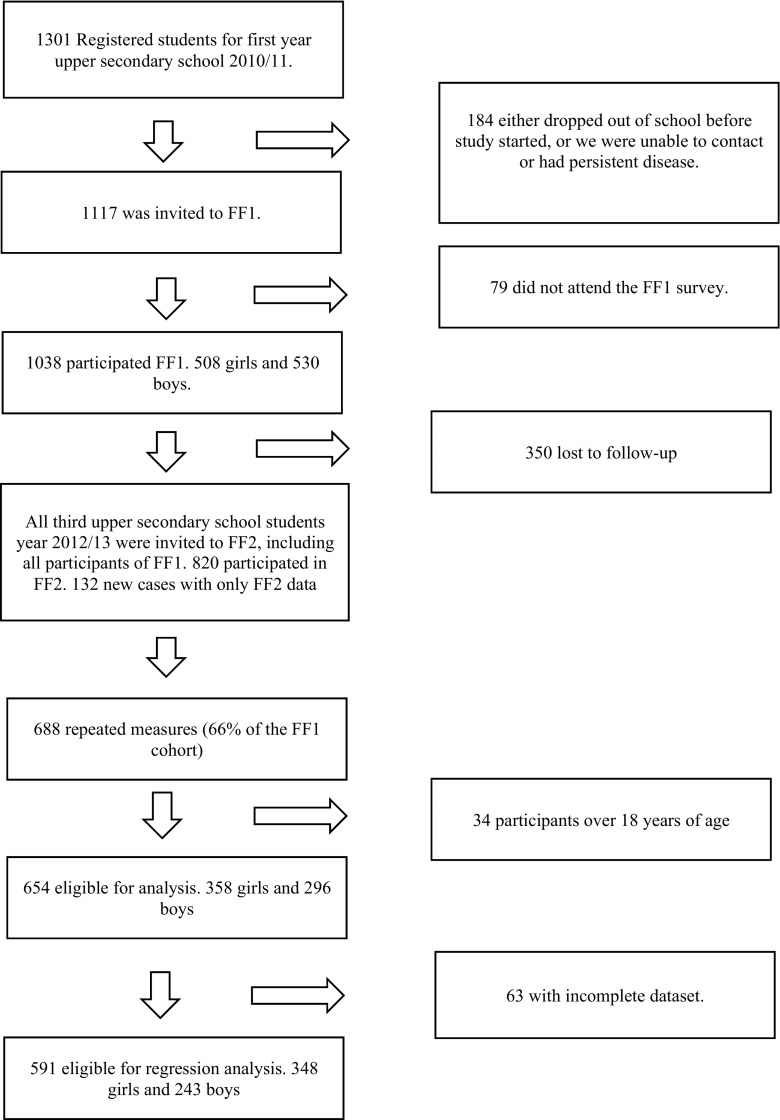
Flowchart of participation in Fit Futures 1 (TFF1) 2010–2011 and Fit Futures 2 (TFF2) 2012–2013

The Tromsø Study is an ongoing population-based epidemiological study with seven repeated surveys conducted among the adult population since 1974 [17]. As part of the Tromsø Study, Fit Futures invited all first year upper secondary school students in Tromsø and the neighboring municipalities to a comprehensive health survey in 2010–2011 (TFF1, baseline). The invited cohort comprised 1117 adolescents and 1038 (508 girls and 530 boys) attended the survey (attendance rate 93%). Among those, 95% of the participants were in the range between 15 and 18 years of age. Two years later, in 2012–2013, all third year upper secondary school students in the same schools and all TFF1 participants not attending school at that time were invited to a follow-up survey, Fit Futures 2 (TFF2). In total, 820 adolescents attended, providing 688 repeated measures of aBMD (66% of the TFF1 cohort) (Fig. 1). The Clinical Research Unit at the University Hospital of North Norway conducted both surveys during school days. The Regional Committee of Medical Research Ethics approved the study (Ref. 2013/1459/REK nord). The study protocol for TFF1 was approved by The Norwegian Data Inspectorate 27.07.2010 (Ref. 07/00886-7/CGN) and the Regional Committee for Medical Research Ethics (REK-Nord) 16.09.2010 (Ref. 2009/1282-23). The study protocol for TFF2 was approved as an extension of the prior approval by the Data Inspectorate 31.10.2012 (Ref. 07/00886-15/EOL). All participants gave written informed consent. Participants below 16 years of age had to bring written consent from their superiors to attend the survey.

### Measurements

We measured total body (TB), total hip (TH), and femoral neck (FN), bone mineral content (BMC; g), bone area (BA; cm^2^), and aBMD (g/cm^2^) by DXA (GE Lunar prodigy) and performed analyses by Encore pediatric software [18]. The densitometer coefficient of variation (CV = [SD/mean] × 100) has been estimated to 1.14% at the total hip measured in vivo [19]. We used the same densitometer in both surveys, and no densitometer drift was detected between the surveys. Trained technicians performed the measurements, and the quality assessment was done according to the same protocol in both surveys. We used measurements of left hip at both femoral sites. In 15 cases, left hip data was missing and the right hip was used. Measurements from the same hip were used in both TFF1 and TFF2. Height and weight were measured to the nearest 0.1 cm and 0.1 kg on the same electronic scale in both surveys (Dong Sahn Jenix, Korea), with participants wearing no shoes and light clothing. We assessed use of medication, acute and chronic diseases, hormonal contraceptive use, and the possibility of pregnancy by clinical interviews, and pregnant participants were excluded from DXA scanning. Participant’s answers on diseases and use of medication known to affect bone were operationalized into dichotomous variables. Hormonal contraceptive use were categorized into no use, combined estrogen and progestogen-based contraceptive (CHC) use, and progestogen-only contraceptive use. We collected sexual maturation information by self-administered questionnaires. In girls, pubertal status was determined through the following questions: “If you have started menstruating, how old were you when you had your first menstruation.” Answers were categorized into “early” (<12.5 years at menarche), “intermediate” (12.5–13.9 years), or “late” (>14 years) sexual maturation. Boys were examined according to Pubertal Developmental Scale (PDS). The boys self-rated secondary sexual characteristics as growth spurt, pubic hair growth, changes in voice, and facial hair growth on a scale from 1 (have not begun) to 4 (completed). We summarized the score and divided by 4. We categorized a score <2 as “have not begun,” 2–2.9 as “barely started,” 3–3.9 as “underway,” and a score of 4 as “completed” [20]. The participants were asked to grade leisure time physical activity (PA) in an average week during the last year according to a four-level scale, which are sedentary activities only; moderate activity like walking, cycling, or exercise at least 4 h per week; participation in recreational sports at least 4 h per week; or participation in hard training/sports competitions several times a week. This question was developed by Saltin and Grimby [21] and has previously been validated in the Tromsø Study [22]. Questions on smoking and snuffing had the following three alternatives: never, sometimes, or daily, while frequency of alcohol consumption had the following five alternatives: “never,” “once per month or less,” “two to four times per month,” “two to three times per week,” and “four or more times per week.” We dichotomized answers on smoking, snuffing, and alcohol into yes and no.

### Statistical analyses

All analyses were performed sex stratified. We calculated means and standard deviations for continuous variables and percentage for categorical variables to describe the study population characteristics. Differences in anthropometric and DXA measures between FF1 and TFF2 were tested using paired sample *t* test, while dichotomous lifestyle factors were tested with McNemar’s test. We explored differences between participants and non-responders in TFF2 using Student’s *t* test and chi-squared testing. Average absolute change and percentage change for BMC and aBMD for each skeletal site were calculated by the difference between the measurements (*T*_2_ − *T*_1_). We used exact measurement dates to compute annual change to account for differences in time between measurements. We stratified participants by age and used one-way ANOVA and multiple comparisons with Bonferroni post hoc test to examine differences in mean aBMD change between groups. We calculated individual age and sex-specific height, weight, FN, TH, and TB aBMD and BMC z-scores (standard deviations away from the sample specific mean) and examined correlations between baseline and follow-up using Pearson’s correlation coefficient. Because height and weight are known determinants of aBMD and the adjustment for height in the two-dimensional DXA scans is incomplete, partial correlation was used to adjust for TFF1 height and weight as well as change in height and weight. We stratified participants into quartiles of aBMD and BMC z-scores and examined the proportions of participants that remained within quartiles, drifted upwards, or drifted downwards between TFF1 and TFF2. Furthermore, an aBMD z-score change variable were computed (*Z*_2_ − *Z*_1_). To test whether baseline age, anthropometric traits (height, weight), and lifestyle factors (PA, alcohol consumption, smoke use, and snuff use) were associated with positive deviation from tracking (z-score change >0), we used logistic regression. The reference categories were no change or downwards drift (z-score change ≤0). Odds ratios (ORs) with 95% confidence intervals (CIs) for upwards drift during follow-up were calculated. We simultaneously adjusted for age, anthropometric measures, lifestyle variables, sexual maturation, and time between measurements. The influence of other relevant confounders like baseline aBMD z-score, ethnicity, chronic disease, and medication known to affect bone health bone and hormonal contraceptive use (girls) were explored, and purposeful selection was used to select final model [23]. We evaluated relevant two-way interactions. We fitted models for FN, TH, and TB separately and ran logistic regression diagnostics, and assumptions were met. Significance level was set to *p* = 0.05 in all analysis, and all procedures were performed in SPSS version 23.

## Results

### Descriptives

We included 654 adolescents, 358 girls and 296 boys aged 15 to 17 at baseline in the present analysis (Table 1). The majority were 16 years of age (*n* = 534), while a small group of 28 participant were 15 years at baseline. Mean follow-up time was 1.94 years (SD 0.20). Thirty-two percent of TFF1 participants were lost to follow-up. Dropout analysis showed statistically significant higher proportion of boys, smokers, snuff users, and consumers of alcohol (girls only) among non-responders compared to those who participated in both surveys.

**Table 1:** Characteristics at baseline survey Fit Futures 1 (TFF1) and follow-up survey Fit Futures 2 (TFF2) 2 years later: continuous variables presented as mean (standard deviation) and categorical variables in percentage

	Girls					Boys				
	TFF1		TFF2			TFF1		TFF2		
	*n*		*n*		*p*	*n*		*n*		*p*
Age	358	16.61 (0.387)	358	18.60 (0.40)		296	16.60 (0.367)	296	18.65 (0.35)	
Age groups at baseline										
15	9	2.5%				19	6.4%			
16	296	82.7%				238	80.4%			
17	53	14.8%				39	13.2%			
Height (cm)	358	165.07 (6.47)	358	165.77 (6.56)	<0.001	296	177.25 (6.52)	296	179.08 (6.49)	<0.001
Weight (kg)	358	60.42 (10.61)	358	63.11 (11.91)	<0.001	296	69.81 (13.68)	296	75.21 (14.64)	<0.001
Sexual maturation^a^										
Early/completed	110	31.3%				22	9.1%			
Intermediate/underway	168	47.9%				177	72.8%			
Late/barely started	73	20.8%				44	18.1%			
Ethnicity										
White	350	97.8%				291	98.3%			
Others	8	2.2%				5	1.7%			
Physical activity										
Sedentary	43	12.0%	47	13.3%		77	26.3%	81	28.4%	
Moderate	141	39.5%	144	40.8%		75	25.6%	60	21.1%	
Sports	110	30.8%	110	31.2%		71	24.2%	77	27.0%	
Competition	63	17.6%	52	14.7%		70	23.9%	67	23.5%	
Smoking (yes)	68	19.0%	102	28.5%	<0.001	62	20.9%	114	38.5%	<0.001
Snuff use (yes)	108	30.2%	152	42.5%	<0.001	108	36.5%	142	48.0%	<0.001
Alcohol consumption (yes)	262	73.2%	336	93.9%	<0.001	195	65.9%	272	91.9%	<0.001
Diseases known to affect bone^b^ (yes)	4	1.1%				5	1.7%			
Medication known to affect bone^c^ (yes)	8	2.2%				6	2.0%			
Hormonal contraceptive use (yes)	118	33.0%								
Estrogen and progestogens	105	29.3%								
Progestogens only	13	3.6%								

^a^Sexual maturation in girls: menarche age. Categories are early (<12.5), intermediate (12.5–13.9), and late (>14). Sexual maturation in boys: Puberty Developmental Scale. Categories are have not begun (<2), barely started (2–2.9), underway (3–3.9), and completed (4)

^b^Diseases known to affect bone (ICD10): E03 hypothyroidism, E10 diabetes type 1, F50.9 eating disorders, K90.0 celiac disease, and M13 arthritis

^c^Medication known to affect bone (ATC): D07A plain corticosteroids, H03A thyroid preparations, N03A antiepileptic, R01AD corticosteroids, R03BA glucocorticoids (inhalants), and H02A corticosteroids for systemic use

### Changes in bone traits and anthropometry

**Fig. 2:**
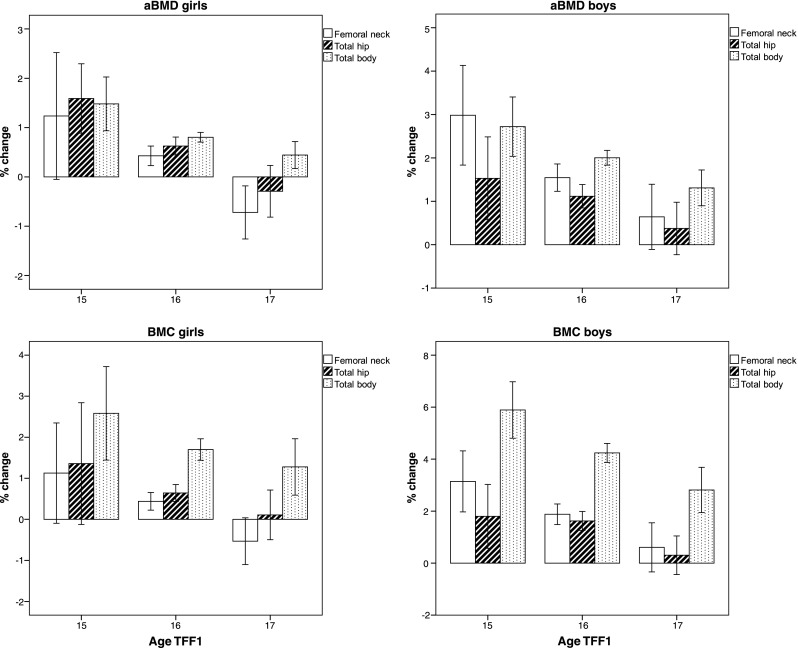
Mean annual percent change in femoral neck total hip and total body aBMD and BMC for girls and boys stratified by age at Fit Futures 1 (TFF1) with 95% confidence intervals

In the overall study, population aBMD increased significantly *(p <* 0.05) at all sites in both sexes. Mean annual percentage increase for FN, TH, and TB aBMD (g/cm^2^) was 0.3, 0.5, and 0.8 in girls and 1.5, 1.1, and 2.0 in boys, respectively (*p* < 0.05). A similar pattern was present for BMC. When stratified into age at baseline, mean annual percent change in aBMD at all skeletal sites decreased successively by increasing age in both sexes (Fig. 2). The differences in annual aBMD changes between age groups were statistically significant (*p* < 0.05) at most skeletal sites and ages; the exceptions were changes in TH aBMD between all age groups and FN aBMD between age 16 and 17 years in boys, as well as changes in FN and TH aBMD between 15- and 16-year-old girls. Girls 17 years of age at TFF1 had a mean annual percentage FN aBMD loss of −0.61 (95% CI −0.15, −1.07) and −0.14 (−0.54, 0.27) at the total hip. Average annual percentage BA change for FN, TH, and TB were 0.01, 0.09, and 2.30 and 0.23, 0.39, and 2.10 for girls and boys, respectively. The average annual height and weight changes during the follow-up period were 0.36 cm (95% CI 0.32–0.41) and 1.37 kg (1.11–1.63) for girls and 0.93 cm (0.83–1.03) and 2.70 kg (2.35–3.04) for boys, respectively.

### Tracking from baseline to follow-up

**Fig. 3:**
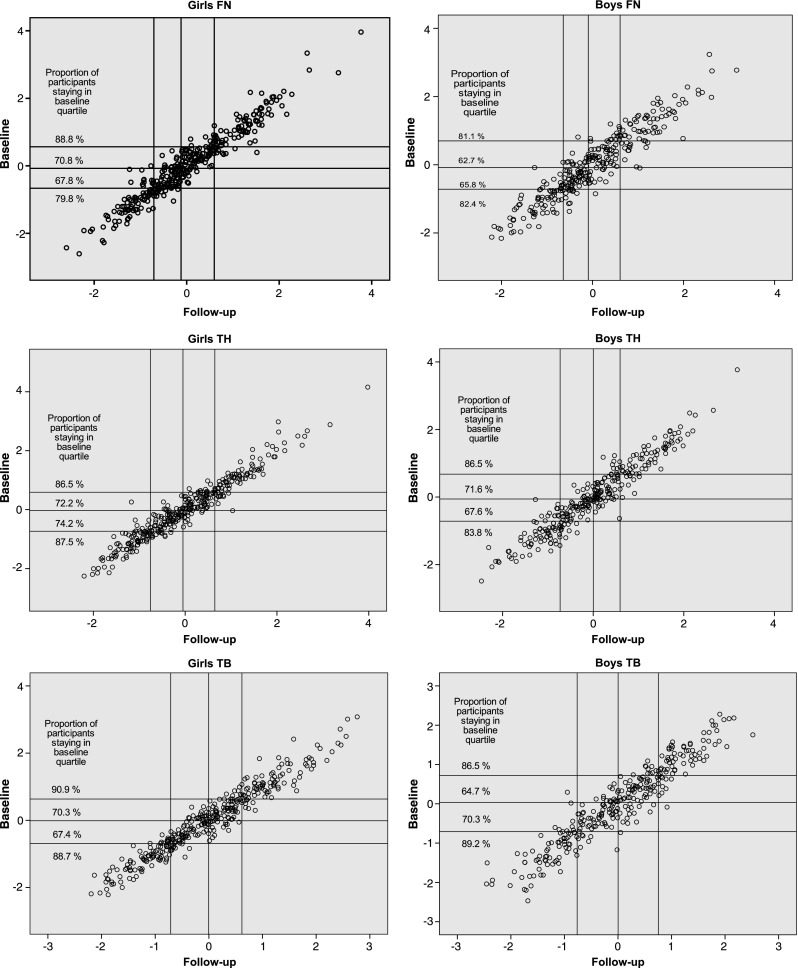
Scatterplot of aBMD z-score for femoral neck (FN), total hip (TH), and total body (TB) at baseline vs z-score at follow-up with proportions of participants remaining in baseline quartile. *Lines* represent the cutoff for percentiles 25, 50, and 75%. Measurements outside diagonal quartiles have changed quartile between baseline and follow-up. Participants were 15–17 years of age at baseline. Boys *n* = 296. Girls *n* = 358

Correlations between TFF1 and TFF2 z-scores were high in both sexes at aBMD FN, TH, and TB, Pearson’s *r* = 0.960, 0.966, and 0.967 for girls and 0.937, 0.955, and 0.946 for boys, respectively. Calculations of coefficients for BMC, height, and weight showed similar strong correlations. Adjusting for TFF1 height and weight or changes in height and weight using partial correlation did not change the aBMD results (not shown). Age-stratified coefficients showed weaker correlation at all sites for 15-year-old boys, FN 0.884, TH 0.871, and TB 0.853 (*N* = 19). All correlation coefficients were statistically significant (*p* < 0.0001). Overall, 78.2% of the girls kept their FN aBMD quartile position between measurements, correspondingly 73% of the boys. The same stability within quartiles was found at TH and TB, 79.6 and 77.4% for girls and 79.2 and 77.7% for boys, respectively. Figure 3 illustrates z-score drift between baseline, and follow-up and shows proportions of participants remaining in each specific quartile.

### Predictors of positive deviation from tracking

Baseline FN, TH, and TB aBMD z-scores had a statistically significant association with lower odds of positive deviation from tracking for both girls and boys (Table 2). Later sexual maturation tended to be associated with higher odds of positive drift at several skeletal sites, with a statistically significant association for TB in girls. For boys, baseline body weight was associated with higher odds of positive deviation at TH (*p* = 0.018), and a statistically significant interaction between age and weight was detected at FN; when stratified into younger (<16.66 years) and older (≥16.67 years) boys, the association between baseline weight and higher odds of positive deviation in FN aBMD was limited to the younger boys (*p* = 0.039). There were no statistically significant associations between lifestyle factors and higher aBMD z-scores in boys; smoking only tended to be associated with decreased odds for higher TH aBMD z-score at follow-up (*p* = 0.062). In girls, snuff and alcohol use were associated with significantly lower odds of higher TH and TB aBMD z-scores, respectively. Also, CHC use was associated with reduced odds of upwards drift during follow-up at FN (*p* = 0.048). Baseline recreational PA level was positively associated with significantly higher TB aBMD z-score at follow-up in girls; participation in recreational sports at least 4 h per week and participation in hard training/sports competitions several times a week were associated with a fourfold and threefold increase in the odds of higher TB aBMD, respectively. Data also indicated a more moderate effect of PA on FN aBMD in girls (*p* = 0.080; Table 3).

**Table 2:** Mean and (standard deviation) of bone traits and time between measurements: areal bone mineral density (aBMD), bone mineral content (BMC), and bone area (BA) for femoral neck (FN), total hip (TH), and total body (TB) at baseline survey Fit Futures 1 (TFF1) and follow-up survey Fit Futures 2 (TFF2) 2 years later

	Girls					Boys				
	TFF1		TFF2			TFF1		TFF2		
	*n*		*n*		*p*	*n*		*n*		*p*
aBMD FN (g/cm^2^)	358	1.07 (0.13)	357	1.08 (0.13)	0.008	296	1.11 (0.15)	296	1.14 (0.15)	<0.001
aBMD TH (g/cm^2^)	357	1.06 (0.13)	357	1.07 (0.13)	<0.001	296	1.12 (0.15)	296	1.14 (0.16)	<0.001
aBMD TB (g/cm^2^)	357	1.14 (0.08)	358	1.16 (0.07)	<0.001	296	1.18 (0.10)	296	1.23 (0.09)	<0.001
BMC FN (g)	358	4.92 (0.71)	357	4.94 (0.72)	<0.001	296	5.99 (0.99)	296	6.19 (0.99)	<0.001
BMC TH (g)	357	32.03 (4.84)	357	32.42 (4.95)	<0.001	296	40.17 (6.64)	296	41.26 (6.86)	<0.001
BMC TB (g)	357	2524.06 (388.27)	358	2600.95 (381.68)	<0.001	296	2963.78 (469.83)	296	3200.96 (476.10)	<0.001
BA FN (cm^2^)	358	4.60 (0.34)	357	4.60 (0.34)	0.866	296	5.38 (0.39)	296	5.41 (0.37)	0.003
BA TH (cm^2^)	357	30.15 (2.32)	357	30.22 (2.38)	0.068	296	35.73 (2.47)	296	35.99 (2.51)	<0.001
BA TB (cm^2^)	357	2207.37 (233.59)	358	2241.68 (224.95)	<0.001	296	2496.46 (240.06)	296	2598.28 (237.87)	<0.001
Time between measurements (years)	358	1.94 (0.20)				296	2.01 (0.23)

**Table 3:** Baseline anthropometric measures and lifestyle factors associated with positive deviation from tracking (z-score change >0) over 2 years in late adolescence

	Girls						Boys					
	FN (*n* = 183 vs 167)		TH (*n* = 182 vs 167)		TB (*n* = 170 vs 180)		FN (*n* = 117 vs 123)		TH (*n* = 127 vs 114)		TB (*n* = 112 vs 129)	
	OR (95% CI)	*p*	OR (95% CI)	*p*	OR (95% CI)	*p*	OR (95% CI)	*p*	OR (95% CI)	*p*	OR (95% CI)	*p*
Age (year)	0.64 (0.35, 1.16)	0.142	*0.43 (0.24, 0.79)*	*0.007*	0.71 (0.39, 1.29)	0.265	^a^		0.56 (0.23, 1.37)	0.205	0.56 (0.23, 1.38)	0.211
Height (cm)	1.00 (0.96, 1.04)	0.855	1.01 (0.97, 1.04)	0.805	1.02 (0.98, 1.06)	0.390	1.00 (0.95, 1.05)	0.988	1.00 (0.95, 1.05)	0.988	*1.06 (1.01, 1.11)*	*0.023*
Weight (10 kg^b^)	1.00 (0.98, 1.03)	0.711	1.00 (0.82, 1.32)	0.744	1.23 (0.93, 1.61)	0.143	^a^		*1.36 (1.05, 1.76)*	*0.018*	1.21 (0.94, 1.57)	0.141
Z-score at baseline	*0.74 (0.58, 0.96)*	*0.022*	*0.74 (0.57, 0.97)*	*0.026*	*0.66 (0.49, 0.88)*	*0.005*	*0.67 (0.47, 0.94)*	*0.021*	*0.64 (0.45, 0.90)*	*0.011*	*0.50 (0.35, 0.72)*	*0.000*
Sexual maturation	Reference: menarche age <12.5 years	Reference: pubertal development completed										
Intermediate/underway	1.41 (0.84, 2.37)	0.198	1.24 (0.73, 2.11)	0.420	1.28 (0.75, 2.18)	0.376	2.55 (0.84, 7.74)	0.098	1.55 (0.56, 4.23)	0.399	1.09 (0.52, 5.87)	0.871
Late/just started	1.68 (0.88, 3.20)	0.114	1.68 (0.87, 3.21)	0.120	*2.05 (1.05, 3.98)*	*0.035*	2.44 (0.67, 8.61)	0.167	1.50 (0.46, 4.90)	0.502	1.74 (0.52, 5.87)	0.371
Hormonal contraceptive use	Reference: no contraceptive use											
Estrogen and progestogen	*0.60 (0.36, 1.00)*	*0.048*										
Progestogen only	0.79 (0.24, 2.55)	0.687										
Physical activity	Reference: sedentary											
Moderate	1.94 (0.93, 4.06)	0.080	1.52 (0.72, 3.21)	0.278	1.78 (0.82, 3.86)	0.147	0.62 (0.27, 1.40)	0.276	0.61 (0.26, 1.39)	0.237	0.53 (0.23, 1.20)	0.128
Sports	1.79 (0.82, 3.90)	0.143	1.64 (0.75, 3.61)	0.217	*4.07 (1.78, 9.30)*	*0.001*	0.56 (0.24, 1.30)	0.138	0.80 (0.34, 1.85)	0.594	0.71 (0.31, 1.62)	0.414
Competition	1.95 (0.80, 4.72)	0.140	1.24 (0.51, 3.01)	0.640	*3.28 (1.31, 8.20)*	*0.011*	0.83 (0.35, 2.00)	0.680	0.70 (0.29, 1.73)	0.443	1.45 (0.62, 3.40)	0.390
Snuff use ^c^	0.93 (0.52, 1.66)	0.802	*0.50 (0.28, 0.89)*	*0.019*	1.09 (0.61, 1.94)	0.769	0.77 (0.35, 1.68)	0.513	0.71 (0.32, 1.54)	0.384	0.61, (0.27, 1.35)	0.221
Smoking ^c^	0.80 (0.42, 1.54)	0.510	1.03 (0.54, 1.99)	0.919	1.17 (0.61, 2.34)	0.645	0.53 (0.22, 1.30)	0.166	0.43 (0.18, 1.04)	0.062	0.47 (0.19, 1.15)	0.097
Alcohol consumption ^c^	1.03 (0.60, 1.78)	0.913	1.24 (0.71, 2.15)	0.450	*0.45 (0.26, 0.80)*	*0.006*	0.87 (0.45, 1.67)	0.674	0.84 (0.43, 1.64)	0.611	1.55 (0.80, 2.99)	0.193

Odds ratios (OR) for femoral neck (FN), total hip (TH), and total body (TB) with confidence intervals (CIs). Reference group were no change or negative deviation from tracking (z-score change ≤0). All the variables are mutually adjusted for other variables in the model including time between measurements. *P* < 0.05 in italics

^a^Significant interaction between age and weight *p* = 0.022. When stratified by younger/older age <16.66 years, ORs for weight were 1.49 (95% CI 1.02, 2.18), *p* = 0.039, *n* = 52 vs 68, and age ≥16.67 years, OR for weight 1.03 (0.67, 1.58), *p* = 0.909, *n* = 71 vs 41

^b^Associations with 10 kg change in body weight

^c^Yes/no

## Discussion

This study presents results from a large population-based cohort of adolescents entering young adulthood. Our results indicate that Norwegian adolescents still accumulate bone mass and increase aBMD between 16 and 18 years of age, although bone acquisition decreases significantly with age at all skeletal sites during these 2 years of follow-up. The results also suggest that girls may be reaching an aBMD plateau at femoral sites between 17 and 19 years of age, even with an indicated reduction of aBMD at femoral neck around the age of 19 compared to 2 years earlier. Consistent with our hypothesis, we report that a stable position within quartiles based on aBMD z-scores is kept over 2 years in late adolescence. Baseline z-scores were consistently associated with lower odds of positive deviation from tracking across all skeletal sites for both sexes. In boys, anthropometric baseline measures appeared to be associated with upwards drift. In girls, several lifestyle factors had statistically significant associations. Particularly, PA tended to be beneficial for TB aBMD.

The decrease in FN aBMD for girls between 17 and 19 years of age is unexpected. However, Berger et al. reported similar findings with an average decrease of aBMD in girls around 20 years of age until stabilization and consolidation [24]. As no specific characteristic in these girls could account for this development like late menarche or intensive physical activity, the relationship between BMC and BA and precision of measurement could explain these findings. According to Sundberg et al. [25], pubertal bone growth is due to increased bone size rather than increased density. aBMD will increase only if BMC increases proportionally more than BA [4]. Elaborative analysis showed that mean FN BA in girls aged 17 years at baseline increased while mean BMC dropped slightly resulting in lower mean aBMD. The decreasing trend of bone acquisition with age is similar at all three sites, and changes in femoral sites seem to drop in advance of total body aBMD. This is consistent with other longitudinal studies [26, 27]. Bachrach et al. found that, for girls, gains in aBMD leveled off in total hip, spine, and whole body already at the age of 14.1, 15.7, and 16.4, respectively. Boys tended to reach plateau at the age of 15.7 in total hip and 17.7 in spine and whole body [28]. Differences in statistical analysis used to localize the age of plateau may explain the slightly earlier age indication compared to our findings. The 2-year developmental difference between boys and girls was present in our cohort as well. Hormonal status influences bone development and PBM depends on biological rather than chronological age [29].

Our tracking results are comparable with other studies [10, 12, 13]. In contrast, Buttazzoni et al. [16] concluded with low sensitivity for childhood bone mass scans to predict PBM. Their study included 65 boys and 56 girls with a time frame of 11 years. With the extensive follow-up period and a mean baseline age of 8 years, this study is not directly comparable to ours. Follow-up during PHV is expected to show reduced correlation, and Kalkwarf et al. reported lower correlations in younger children than in older [10]. In our cohort, aBMD tracking for boys became successively stronger as annual height change reduced gradually between 15 and 17 years of age at baseline, indicating this link between statural growth and aBMD tracking (data not shown). The tendency of stronger degree of tracking with cessation of growth strengthens the notion that measures in our study potentially can predict adult bone mineral status. The results for participants in the lowest quartile are of clinical importance and highlight the great challenge of changing the bone mineral-level trajectory of this group. Even though this study has a narrow time span, the fact that a large proportion of adolescents with low bone mass levels remains low supports the hypothesis that subjects susceptible to relatively early osteoporosis risk may be detectable early in life.

The importance of PBM makes it interesting to explore modifiable factors with the potential of altering the bone mass trajectory. Our study suggested that baseline body weight may influence aBMD at femoral sites in boys, but not in girls. Age being an effect modifier of weight for boys at FN is biologically reasonable because bone adaptation to mechanical loading is greater in a growing skeleton and FN is highly exposed to weight [30]. No associations between lifestyle factors and positive drift were detected for boys. For girls, associations were incoherent both in terms of direction, statistical significance, and skeletal sites. PA seemed beneficial for TB aBMD, but we found no clear dose-response effect. This may indicate that participants reporting to be in the hard training and competition category at baseline were already at the tail of the z-score distribution as reported by Winther et al. [31]. Sustained activity level during follow-up and preservation of high z-score could lead to classification into the reference group no change or downwards drift for these participants. Previous studies report tobacco use to have a duration and dose-dependent negative effect on aBMD, while the impact of alcohol is more unclear [32–36]. Snuff use and smoking mainly prevented subjects from positive deviation in our study, although not statistically significant at all skeletal sites. However, changes in exposure variables during follow-up make the interpretations of associations challenging. Proportions of smokers, snuff users, and participants consuming alcohol all increased during follow-up (Table 1). The relationship between hormonal contraceptive use and aBMD development remains controversial. Our results indicated CHC use to be disadvantageous for the FN and supports evidence suggesting that CHC use is likely to impair acquisition of optimal PBM [37]. Recent reviews emphasize the need for randomized controlled trials to confirm these effects [38]. Progestogen-only contraceptives have also been associated with reduced aBMD when used before the achievement of PBM [39]. This association was not confirmed in our cohort, but participants reporting to use progestogen-only contraceptives were few. The underlying mechanisms behind the effects of contraceptives are complex and data on length of use and dosage are lacking. Winter et al. reported that late sexual maturation was associated with low aBMD levels in TFF1 [31]. The fact that proportions of sexual maturation categories in our study are comparable with other Norwegian youth cohorts [40] and that the association between late sexual maturation and increased odds for positive deviation in this longitudinal study is consistent suggest that this adverse effect levels out to some extent. As reported by previous studies [10, 12], baseline aBMD z-score appears to be highly predictive of future z-score. The consistent association between high baseline z-score and reduced odds of positive deviation could be due to the phenomenon regression towards the mean. Extreme measures at the tails of the distribution will when repeated tend to be less extreme and closer to average because of variation within the individual or measurement error [41].

The longitudinal design and the large representative sample are among the strengths of the study. The sample has well-described characteristics, is homogenous in age and ethnicity, and included both sexes and participants from both rural and urban regions. We used the same densitometer through both surveys with continuous validations. A well-established research unit ensured high quality of data acquisition. There are, however, limitations to be discussed. Firstly, DXA and aBMD measurements have their limitations. Interpretation of DXA measures of growing skeletons could be problematic because it is a two-dimensional measure and size dependent [42]. aBMD is furthermore only a surrogate measure of bone strength, and the broad concept of PBM captures other parameters like architecture, geometry, and distribution of trabecular and cortical bone [6]. Secondly, non-participation and loss to follow-up could be a problem if only the healthy part of the population chooses to participate. Fourteen percent of the eligible population were not invited because we were unable to get in contact with them due to chronic illness or dropout from school. School dropouts tend be associated with an unhealthy lifestyle [35]. The detected differences in characteristics between non-responders and participants attending both surveys may cause bias. A higher proportion of snuff user among non-responders would make the statistically significant association between snuff use and lower odds for positive drift for girls an underestimation. Thirdly, we acknowledge that the follow-up time of 2 years may be a limitation. Changes over such a short time period are at risk of being obscured by variability in DXA measurements. On the other hand, the recommended minimum interval between DXA scans is 6–12 months [42], and our findings are in accordance with previous reports.

In conclusion, this study corroborates the findings of previous research exploring the dynamics of bone mineral levels in adolescence. We report a high degree of tracking of aBMD levels over 2 years in late adolescence. Because of the short time span between measurements, a longer follow-up is necessary for definite conclusions on tracking. Baseline aBMD z-score was the only consistent predictor of deviation from tracking in both girls and boys. For boys, baseline body weight tended to be associated with upwards drift in aBMD z-score at femoral sites. For girls, lifestyle factors such as PA, snuff use, and consumption of alcohol appeared important, but not persistently across skeletal sites. Further studies are needed in order to investigate the possible effect of changes in anthropometrics and lifestyle factors on development of aBMD in adolescence. Additional follow-up surveys of the Fit Futures cohort are required to explore further longitudinal effects.
